# Edema related to treatment with psychotropic drugs

**DOI:** 10.1007/s00702-024-02738-6

**Published:** 2024-02-14

**Authors:** Johanna Engel, Beatrice Haack, Oliver Zolk, Timo Greiner, Martin Heinze, Sermin Toto, Johanna Seifert, Stefan Bleich, Catherine Glocker, Renate Grohmann, Michael Schneider, Susanne Stübner

**Affiliations:** 1 Brandenburg Medical School, University Clinic for Psychiatry and Psychotherapy, Immanuel Klinik Rüdersdorf, Seebad 82/83, 15562 Rüdersdorf bei Berlin, Germany; 2 Institute of Clinical Pharmacology of the Brandenburg Medical School, Immanuel Klinik Rüdersdorf, Seebad 82/83, 15562 Rüdersdorf bei Berlin, Germany; 3 Department of Psychiatry, Social Psychiatry, and Psychotherapy, Hannover Medical School, Carl-Neuberg-Straße 1, 30625 Hannover, Germany; 4 Department of Psychiatry and Psychotherapy, Ludwig Maximilian University of Munich, Nussbaumstr. 7, 80336 Munich, Germany; 5 Maßregelvollzugsleitung, Klinik für Forensische Psychiatrie, Bezirksklinikum Ansbach, Ludwig-Maximilians-Universität München, Feuchtwanger Straße 38, 91522 Ansbach, Germany

**Keywords:** Edema, Adverse drug reaction, AMSP program, Psychotropic drugs, Pregabalin, Mirtazapine

## Abstract

Edema as an adverse drug reaction is a commonly underestimated yet potentially debilitating condition. This study analyzes the incidence of severe psychotropic drug-induced edema (e.g., edema affecting the face, legs, or multiple body parts and lasting for more than 1 week, or in any case necessitating subsequent diuretic use) among psychiatric inpatients. The cases under examination are derived from an observational pharmacovigilance program conducted in German-speaking countries (“Arzneimittelsicherheit in der Psychiatrie”, AMSP) from 1993 to 2016. Among the 462,661 inpatients monitored, severe edema was reported in 231 cases, resulting in an incidence of 0.05%. Edema occurred more frequently in women (80% of all cases) and older patients (mean age 51.8 years). Pregabalin had the highest incidence of severe edema, affecting 1.46‰ of patients treated with pregabalin, followed by mirtazapine (0.8‰). The majority of edema cases showed a positive response to appropriate countermeasures, such as dose reduction and drug discontinuation, and resolved by the end of the observation period. While most instances of drug-induced edema are reversible, they can have a significant impact on patient well-being and potentially result in decreased treatment adherence. It is, therefore, crucial to remain vigilant regarding risk-increasing circumstances during treatment with psychotropic drugs.

## Introduction

Edema is characterized by the accumulation of fluid in the interstitial space typically caused by either an increase in hydrostatic pressure, a decrease in colloid osmotic pressure, increased capillary permeability, or impaired lymphatic drainage [Whiting and McCready (2016); Trayes et al. (2013)].

As an adverse drug reaction (ADR) caused by psychotropic drugs, it is not uncommon and often causes discomfort to those affected (Umar and Abdullahi 2016). Given that edema often resolves on its own, its incidence is likely underestimated, as patients may not always report it, and non-severe manifestations may be inadvertently overlooked by healthcare providers. Additionally, the multifactorial nature of its etiology can make pinpointing the exact cause challenging (Largeau et al. 2021). Nevertheless, the substantial negative impact on patients’ well-being and, consequently, their adherence to treatment are a matter of significant concern.

Among antipsychotics, edema is most commonly reported in patients treated with olanzapine (Nayak et al. 2009), quetiapine (Polat et al. 2019), and risperidone (Obayi 2018), as well as ziprasidone (Ku et al. 2006) and amisulpride (Chen and Chou 2004). Among the antidepressants, cases have been reported for mirtazapine (Lahdelma and Bruin 1998), trazodone (Barrnett et al. 1985), and citalopram (Ravi et al. 2014). However, data on the incidence of edema during psychotropic drug treatment vary widely.

Reported incidences range from 1 to 10% for olanzapine (European Medicines Agency 2010), quetiapine (European Medicines Agency 2014), mirtazapine (European Medicines Agency 2008), and risperidone (European Medicines Agency 2022). In a systematic review on the incidence of antipsychotic drug-related edema, Umar and colleagues examined 30 studies that reported edema and found a prevalence of 29.4% each in patients treated with risperidone, olanzapine, and quetiapine (Umar and Abdullahi 2016). On the other hand, Ng and colleagues (2003) reported a significantly higher incidence of 57% for olanzapine; however, these data were based on only 49 patients, which had also some somatic comorbidities. While increased vascular hydrostatic pressure is often considered a primary contributing factor, the exact mechanism of psychotropic drug-induced edema remains elusive (Largeau et al. 2021). Several hypotheses have been postulated, although not all proposed mechanisms necessarily apply to the psychotropic drugs that are frequently associated with drug-induced edema. One possible explanation may involve blockade of alpha-1-receptors, which causes peripheral vasodilation, thereby promoting the development of edema (Ng et al. 2003). However, this mechanism of action does not apply to all psychotropic drugs, that have been associated with drug-induced edema. For example, the commonly implicated drug mirtazapine does not exhibit a high affinity for this receptor. Another hypothesis suggests an allergic reactions as a potential cause. Thus, Ku and colleagues (2006) found increased Ig-E titers and moderately elevated C3 and C4 titers in the plasma of patients treated with ziprasidone. This hypothesis is supported by in vivo experiments in mice showing that histamine can increase vascular permeability (Ashina et al. 2015). Interestingly, Munari and colleagues 2015 showed that the administration of citalopram increased extraneuronal histamine levels in mice. They also revealed a reciprocal influence of serotonin and histamine, as histamine also increased serotonin release by binding to histamine receptors subtype 1 (H1) in the raphe nuclei.

A further theory suggests that the blockade of 5-hydroxytryptamine receptor 2 (5-HT2) results in vascular muscle relaxation (Ng et al. 2003), a phenomenon also observed in idiopathic edema (Kuchel et al. 1975). Another possible explanation is a decrease in the concentration of second messengers by binding to 5-HT2-receptors, muscarinic acetylcholine receptors subtype 1 (M1) and H1-receptors, resulting in reduced vascular contractility (Wustmann et al. 2009). Although the exact pathomechanisms are not yet fully understood, several studies suggest that older age and female sex appear to be associated with an increased risk of edema [Umar and Abdullahi (2016); Obayi (2018)]. Comorbidities, such as diabetes mellitus, obesity, arterial hypertension, congestive heart failure, and chronic venous disease, have also been linked to the occurrence of drug-induced edema (Largeau et al. (2021); Robertson et al. (2004)). Lastly, psychotropic drug-induced renal impairment can lead to increased water retention, subsequently elevating hydrostatic pressure and contributing to development of edema [Bodenstein and Lavin (2022); Cho and Edwin (2002)].

The aim of our study is to compare psychotropic drugs in terms of their risk for the occurrence of edema and to investigate possible risk factors, such as age, sex, and comorbidities.

## Methods

### The AMSP program

This study is based on the AMSP (German: “Arzneimittelsicherheit in der Psychiatrie”, “drug safety in psychiatry”) drug surveillance program, which has been continuously documenting the occurrence of severe ADRs among psychiatric inpatients within the naturalistic clinical setting, primarily in Germany, Austria, and Switzerland, since 1993. In accordance with the AMSP protocol, an ADR is classified as severe and included in the study if it meets one of the following criteria: (1) it poses a potentially life-threatening risk or severely endangers health, (2) it impairs the ability to perform activities of daily living, or (3) it necessitates specialized care by another department or physician. The AMSP protocol provides additional guidelines specific to each organ system to further standardize the classification process (Grohmann et al. 2014).

### Assessment and collection of ADRs

The AMSP program comprises two datasets. The first dataset contains information related to psychotropic drug use within the inpatient psychiatric setting of the participating hospitals. Data collection occurs half-yearly in participating hospitals, which provides information on the number of patients treated annually, drug use data including dosage of all drugs administered, as well as basic sociodemographic patient characteristics and diagnoses. The second dataset includes information on the occurrence of ADRs. ADRs are documented by drug monitors (usually physicians), who have been alerted to ADR occurring in a patient on a psychiatric ward. Drug monitors routinely inquire (i.e., biweekly) with treating physicians regarding the incidence of ADRs. A standardized questionnaire is used, including detailed information on the ADR, all drugs used during onset of the ADR, onset and progression of the ADR, previous exposure to the imputed drugs, drug treatment after the ADR, duration of the treatment and outcome in the event of rechallenge. Cases are discussed with attending physicians and then sent to regional centers for secondary evaluation. A selection of cases is then discussed at regional and central case conferences which are attended by the drug monitors from participating hospitals, drug safety representatives of pharmaceutical companies supporting the AMSP project, as well as representatives of the Federal Institute for Drugs and Medicinal Devices (Bundesinstitut für Arzneimittel und Medizinprodukte) and the Drug Commission of the German Medical Association (Arzneimittelkommission der deutschen Ärzteschaft). For each drug administered to a patient experiencing an ADR, a probability rating is assigned, which can be subject to revision during case conferences. The probability rating is categorized as follows:

- Grade 1 *(possible)*: ADR not known, time course or dosage unusual for drug in question, or alternative explanation for adverse event more probable
- Grade 2 *(probable)*: ADR known for drug in question, time course and dosage in accordance with previous experience, and alternative explanation less probable
- Grade 3 *(definite)*: In addition to criteria necessary for a probable rating, reappearance of the ADR after rechallenge with drug in question
- Grade 4: ADR improbable but cannot be ruled out definitely or there is a lack of information.

One or more drugs can be implicated in the ADR in question (Grohmann et al. 2004). The following study includes all cases of psychotropic drug-induced severe edema between 1993 and 2016 with “probable” or “definite” causality in the AMSP databank. Because comorbidities and non-psychotropic drugs were documented starting in 2007, only data from 2007 to 2016 can be included in this analysis.

### Definition of edema and drug classification

AMSP classifies edema as a severe ADR if it persists for more than 1 week and affects the limbs or the face or multiple body parts and in any case if the patient is treated with diuretics as a consequence of edema. Two circumstances are considered for the causal attribution of administered drugs: First, ADR in which a single drug is considered to be responsible or, second, ADRS implicating more than one drug. This approach is based on the frequent polypharmacy and possible synergistic effects or interactions of the drugs. The various drugs are assigned to the following groups:

- Antipsychotics: First- and second-generation antipsychotics (FGAs and SGAs),
  FGAS are further subclassified as high- and low-potency
- Antidepressants: Selective serotonin reuptake inhibitors (SSRIs), selective serotonin-norepinephrine reuptake inhibitors (SSNRIs), tricyclic antidepressants (TCAs), noradrenergic and specific serotonergic antidepressants (NaSSAs), and monoamine oxidase inhibitors (MAO-Is)
- Anticonvulsants
- Lithium.

Cases are reported in absolute numbers, providing the exact count of cases and per thousand, which is a relative measure, calculated in relation to the number of patients treated with a specific psychotropic drug during the observation period. Psychiatric diagnoses are reported in accordance with the International Classification of Diseases, 10th Revision (ICD-10).

### Statistical analysis

The incidence of severe edema was assessed in relation to the number of patients exposed to specific drug classes, subclasses, and single drugs. The results are reported with a 95% confidence interval (CI) to provide a measure of the uncertainty in the findings. Due to the relatively low rate of edema, only drugs used in the treatment of 5000 or more patients were included in the statistical analysis. Comparisons of edema rates related to diagnosis, sex, and age (i.e., $\le 30$ years, 30–60 years, and $\ge 60$ years) were performed using Chi-square tests. The significance level was set at *p*$<0.05$. Median and range were calculated for the dosages of the psychotropic drugs imputed alone more than ten times.

### Ethics review

Evaluations based on the AMSP database have been approved by the Ethics Committee of the University of Munich and the Ethics Committee of the Hannover Medical School (Nr. 8100_BO_S_2018). This study adheres to the Declaration of Helsinki and its later amendments. The AMSP program is a continuous observational post-marketing drug surveillance program and does not interfere with the ongoing clinical treatment of the patients under surveillance. Furthermore, evaluation data were extracted from an anonymized data bank, ensuring the privacy and confidentiality of individual patients, and thus, no specific patient can be identified or traced.

## Results

### Population characteristics

Between 1993 and 2016, the AMSP program monitored 495,615 psychiatric inpatients. Out of these 462,661 inpatients (56% female, 44% male) were treated with psychotropic drugs (93.4%). A total of 231 cases of severe edema were documented during this time period, affecting 0.05% of patients. Table 1 shows diagnoses, and sex and age groups of the patients with edema in comparison to all patients treated with psychotropic drugs. More than half of the patients with psychotropic drug-induced edema suffered from depressive disorders (51%), followed by patients with schizophrenia (23%). Women were affected more often than men (80.1 vs. 19.9%; $p<$0.01). The average age of patients with severe edema was 51.8 years (range 20 to 88 years). A significant difference in age, relative to the number of all patients treated, was found in patients under 30 years old ($p<$0.01) as well as in patients 30–60 of age ($p<$0.01), but not in patients over 60 years of age ($p<$0.638).

**Table 1:** Sex, age, and diagnosis of inpatients affected by severe edema

	Monitored inpatients		cases of edema		% of patients	$\chi ^2,p$
	*n* (% of 462 661)		*n* (% of 231)		exposed	
Sex						
Male	204 071	(44.1)	46	(19.9)	0.02	54.8
Female	258 590	(55.9)	185	(80.1)	0.07	<0.01
Age						
$\le 30$	80 568	(17.4)	12	(5.2)	0.01	21.2
30–60	25 8241	(55.8)	154	(66.7)	0.06	<0.01
$\ge 60$	123 852	(26.8)	65	(28.1)	0.05	
Diagnosis						
Affective disorders Depression (F31.3–38.8)	157 998	(34.1)	118	(51.1)	0.07	51.2
Mania (F30 + F31.0–F31.2)	13 167	(2.8)	18	(7.8)	0.14	<0.01
Schizophrenia (F20-29)	158 037	(34.2)	54	(23.4)	0.03	
“Neuroses” and Personality Disorders (F40-48 + F60-62)	56 419	(12.2)	4	(1.7)	0.05	
Addiction (F10-F19)	20 637	(4.5)	8	(3.5)	0.04	
Organic Disorder (F00-09 + F70-F79)	49 029	(10.2)	25	(10.8)	0.01	
Other (F90-98)	7 374	(1.6)	4	(1.7)	0.05	

### Localization and course of edema

Table 2 provides an overview of the location of edema. Edema of the legs was observed in 184 cases, with 126 cases (55.0%) presenting as the sole affected body part. The face was affected in a total of 77 (33.3%) cases, with edema was confined solely to the face in 37 patients (16.0%). The arms were affected in 42 cases, with solitary manifestation in only 3 cases (1.3%). Only one patient experienced edema of the trunk who also had edema of both the face and arms as well as the legs. Edema occurred in more than one body part in 64 patients (27.7%).

**Table 2:** Localization of edema

Body part	Frequency	Only on this part of the body
Arms	42	3
Face	77	37
Legs	184	126
Trunk	1	0

The most common applied countermeasures were discontinuation of the suspected drugs in 195 cases (84.4%), dose reduction in 55 cases (16.1%), and use of diuretics in 66 cases (28.6%). Due to the imputation of more than one drug, both reduction and discontinuation were performed as countermeasures in 29 cases (12.5%).

The mean duration of edema was 16.8 days (standard deviation 5.5 days) and with a median of 11 days (range: 1–252 days). At the end of the observation period, 186 cases of edema had completely resolved (80.5%), 39 were in remission (16.9%), and in 3 cases, the edema remained unchanged (1.3%). The outcome for 3 patients were not reported (1.3%).

### Severe cases of psychotropic drug-induced edema

Several patients had particularly severe manifestations of edematous swelling with significant consequences. Five patients had to be transferred to a general medicine ward due to the widespread edema and its severity of edema to rule out deep leg vein thrombosis. In two instances, one of which resulted in an emergency department visit, the ADR was associated with dyspnea, which fully resolved after discontinuation of the imputed drugs. In six cases, massive swelling of the face resulted in difficulty in opening the eyes. These patients were also more likely to report distress, as the swelling was perceived as “disfiguring”. One case implicating valproate as “probable” cause resulted in swelling of the genitals in addition to swelling of the arms and legs. Among cases of leg edema, the occurrence of stasis dermatitis was explicitly documented in three cases, while one patient developed a secondary erysipelas. In addition, skin rashes and exanthema occurred in four cases. Significant swelling and circumferential enlargement of the legs or hands resulted in eight cases in limited movement.

### Edema risk among psychotropic drugs

**Fig. 1:**
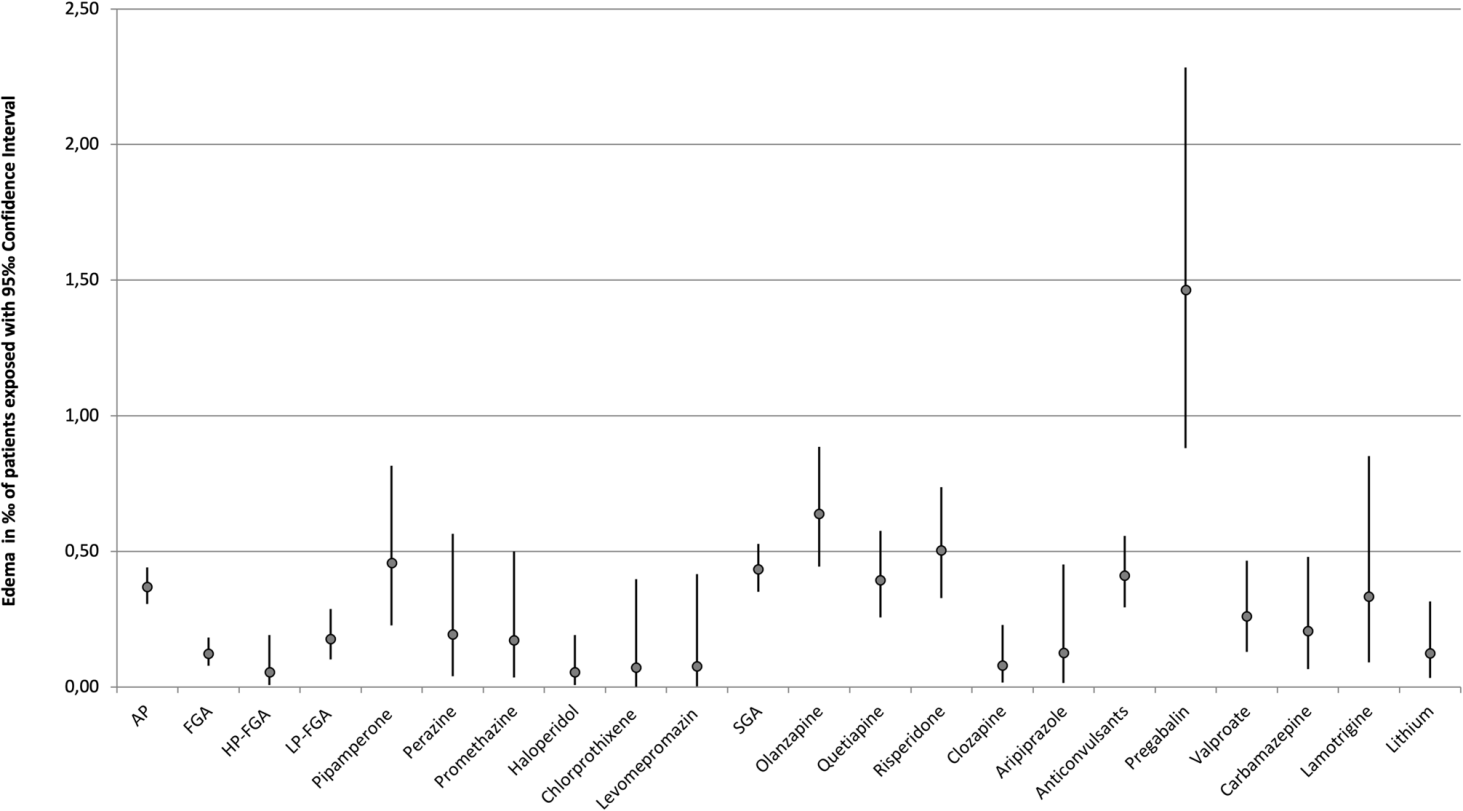
Incidence of edema including 95% CI of antipsychotics and anticonvulsants. Only drugs used in more than 5000 patients and related to at least one case of edema are depicted

**Fig. 2:**
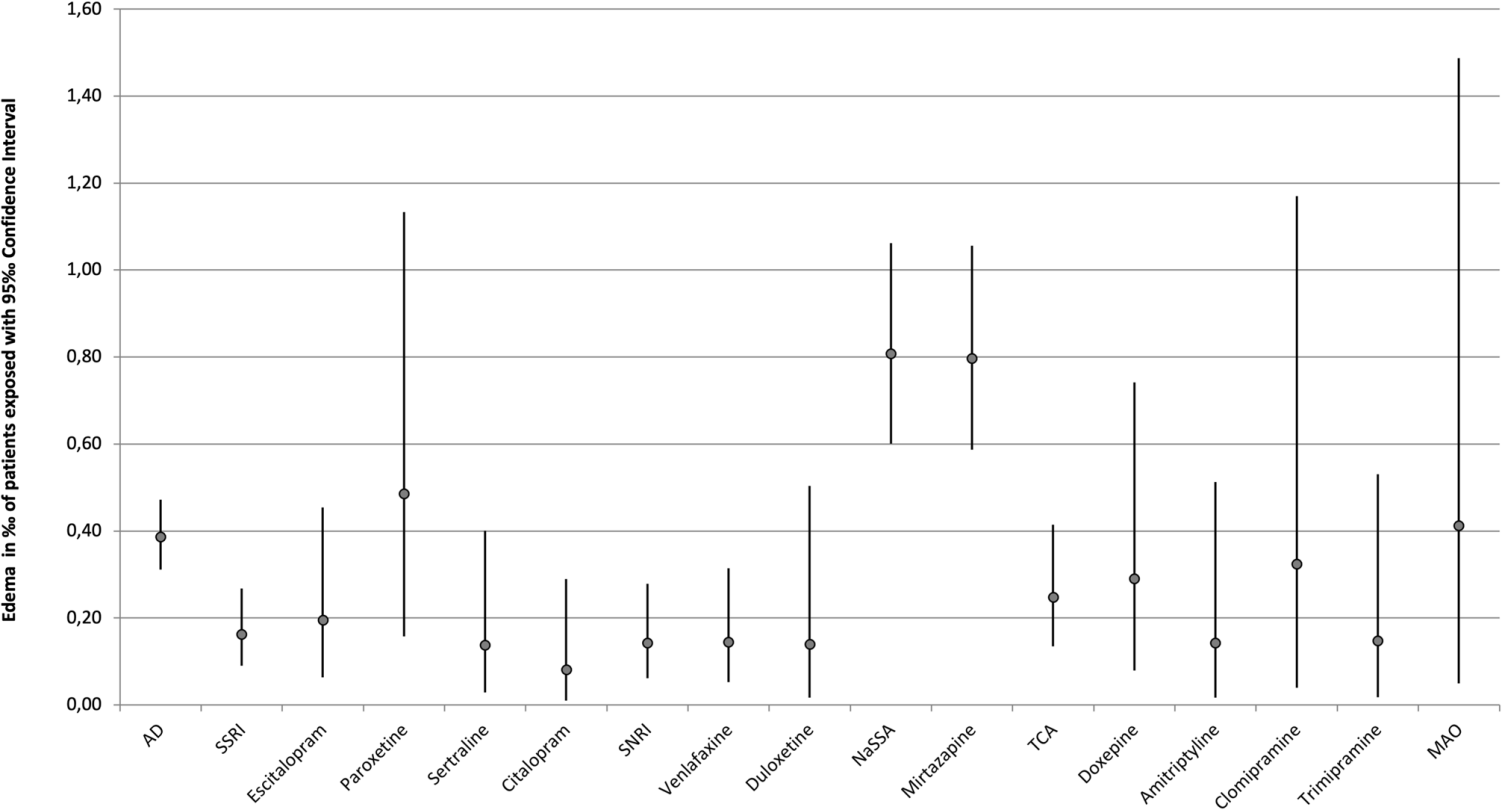
Incidence of edema including 95% CI of antidepressants. Only drugs used in more than 5000 patients and related to at least one case of edema are depicted

The frequency of occurrence of severe edema during treatment with psychotropic drugs is detailed in Table 3. A single drug was imputed as “probable” or “definite” in 201 cases (87.0%), two drugs were imputed in 21 cases (9.1%), and three or more drugs were imputed to cause ADR in 9 cases (3.9%). In addition to the main drug with “probable” imputation, other psychotropic drugs were reported as a “possible” (e.g., due to an unusual time course or because edema is an unusual ADR for the drug in question) cause in 48 cases (20.8%). Antipsychotics were most commonly imputed accounting for 52.8% (*n*=122) of all cases, followed by antidepressants (40.7%, *n*=94). Edema therefore occurred in 0.39‰  of all patients treated with antidepressants and in 0.37‰  of all patients treated with antipsychotics. Anticonvulsants were implicated in 17.7% (*n*=41) of cases occurring in 0.41 ‰ of patients treated with this drug group. The most frequently imputed drugs were mirtazapine (20.8%), olanzapine (14.7%), quetiapine (11.3%), risperidone (11.3%), and pregabalin (8.2%) (Table 3). The rate of severe edema of psychotropic drugs used in less than 5000 patients can be found in Table 7. Pregabalin had the highest overall incidence (1.46‰)(Fig. 1). Among antidepressants mirtazapine had the highest incidence (0.8 ‰) (Fig. 2), while among antipsychotics, olanzapine was most commonly implicated (0.6‰)(Fig. 1). However, it must be pointed out that confidence intervals are wide and partially overlapping (Figs. 1, 2). It is noteworthy that, due to the frequency of mirtazapine’s imputation of edema, NaSSAs were the most frequently imputed subgroup within antidepressants, accounting for 18.1% of cases. Among antipsychotics, SGAs were significantly more often associated with edema in comparison to FGAs (36.2 vs. 8.9%).

**Table 3:** Imputed drug groups of all inpatients and those suffering from edema

Drug group	Monitored inpatients, *n*	Cases of edema, *n*	Incidence in patients exposed ‰	Cases of edema, *n* (imputed alone)	Incidence in patients exposed ‰ (imputed alone)
Antidepressants	243,588	94	0.39‰	72	0.30‰
SSRI	92,496	15	0.16‰	9	0.10‰
Escitalopram	25,667	5	0.19‰	2	0.08‰
Paroxetine	10,298	5	0.49‰	3	0.29‰
Sertraline	21,868	3	0.14‰	2	0.09‰
Citalopram	24,904	2	0.08‰	2	0.08‰
SSNRI	56,530	8	0.14‰	6	0.11 ‰
Venlafaxine	41,559	6	0.14‰	4	0.10‰
Duloxetine	14,343	2	0.14‰	2	0.14‰
NaSSA	63,182	51	0.81‰	40	0.63‰
Mirtazapine	60,305	48	0.80‰	37	0.61‰
TCA	56,666	14	0.25‰	12	0.21‰
Doxepin	13,811	4	0.29‰	4	0.29‰
Amitriptyline	14,089	2	0.14‰	2	0.14‰
Clomipramine	6174	2	0.32‰	2	0.32‰
Trimipramine	13,604	2	0.15‰	1	0.07‰
MAO	4855	2	0.41‰	2	0.41‰
Other	24,761	0	0.00‰	0	0.00‰
Antipsychotics	333,175	122	0.37‰	92	0.82‰
FGA	195,448	24	0.12‰	11	0.06‰
High-potency	37,650	2	0.05‰	2	0.05‰
Haloperidole	37,650	2	0.05‰	2	0.05‰
Low-Potency	90,406	16	0.18‰	6	0.07‰
Pipamperone	24,117	11	0.46‰	6	0.25‰
Perazine	15,495	3	0.19‰	2	0.13‰
Promethazine	17,546	3	0.17‰	0	0.00‰
Chlorprothixene	14,017	1	0.07‰	0	0.00‰
Levomepromazine	13,374	1	0.07‰	1	0.07‰
SGA	226,161	98	0.43‰	80	0.35‰
Olanzapine	54,882	34	0.62‰	30	0.55‰
Quetiapine	66,209	26	0.39‰	20	0.30‰
Risperidone	51,683	26	0.50‰	20	0.39‰
Clozapine	38,349	3	0.08‰	3	0.08‰
Aripiprazole	15,988	2	0.13‰	2	0.13‰
Anticonvulsants	99,846	41	0.41‰	31	0.31‰
Pregabalin	12,984	19	1.46‰	14	1.08‰
Valproate	42,259	11	0.26‰	7	0.17‰
Carbamazepine	24,308	5	0.21‰	4	0.16‰
Lamotrigine	12,023	4	0.33‰	4	0.33‰
Lithium	32,373	4	0.12‰	2	0.06‰

*FGA* first-generation antipsychotic, *MAO* monoamino oxidase inhibitor, *NaSSA* noradrenergic and specific serotonergic antidepressant, *SGA* second-generation antipsychotic, *SSNRI* selective serotonin-noradrenalin reuptake inhibitor, *SSRI* selective serotonin reuptake inhibitor, *TCA* tricyclic antidepressant

Substances that have been applied more than 5000 times but did not cause edema: Flupentixole, Melperone, Zuclopentixole, Prothipendyle, Amisulpride, Diazepam, Lorazepam, Zopiclone, Zolpidem, Biperiden

#### Cases imputing a single drug

**Fig. 3:**
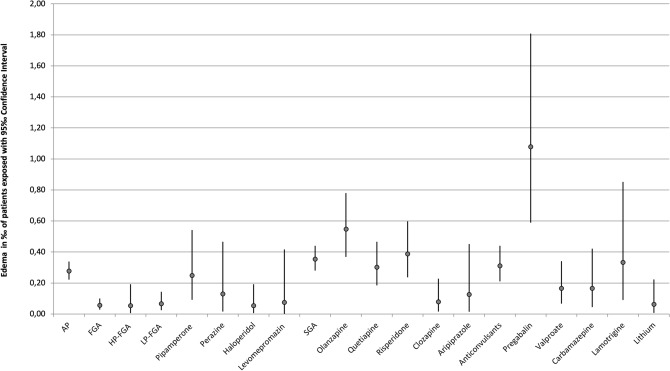
Incidence of edema including 95% CI of antipsychotics and anticonvulsants imputed alone. Only drugs used in more than 5000 patients and related to at least one case of edema are depicted

**Fig. 4:**
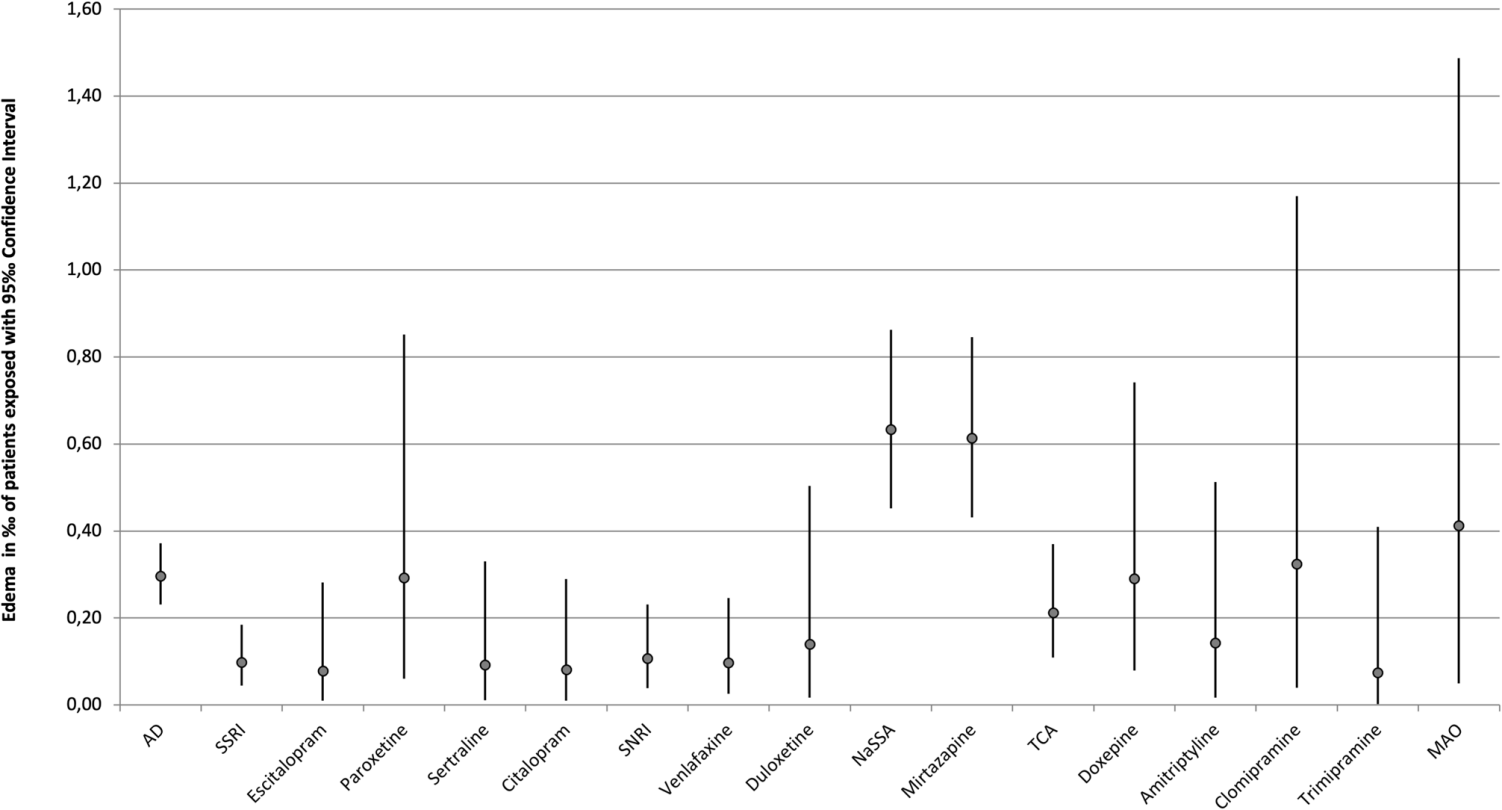
Incidence of edema including 95 % CI of antidepressants imputed alone. Only drugs used in more than 5000 patients and related to at least one case of edema are depicted

Among the 201 cases implicating a single drug, antipsychotics accounted for 92 cases (45.8% of all single imputations), antidepressants for 72 cases (35.8%), and anticonvulsants for 31 cases (15.4%). The corresponding incidence of edema for antipsychotics was 0.82‰ (Table 3, Fig. 3), 0.30‰  for antidepressants (Table 3, Fig. 4) and 0.31‰  for anticonvulsants (Table 3, Fig. 3). Regarding the individual psychotropic drugs, mirtazapine was implicated alone in 37 cases, followed by olanzapine in 30 cases, quetiapine and risperidone in 20 cases each and pregabalin in 14 cases. Of the cases imputing a single psychotropic drug, pregabalin had the highest incidence at 1.08‰  (Table 3, Fig. 3). The dosages (median and range) of the psychotropic drugs that were imputed more frequently than ten times alone are shown in Table 4. For mirtazapine, olanzapine, and quetiapine, the median dosages of the groups of patients with severe edema and monitored inpatients did not differ significantly. For risperidone (2.75 vs. 3 mg) and pregabalin (175 vs. 200 mg), the median dosages were lower in the edema group.

**Table 4:** Median and range of dosages of the psychotropic drugs that have been imputed alone more than ten times

Substance	Monitored inpatients, median (range)		Cases of edema, median (range)	
Mirtazapine	30 mg	(7.5–300 mg)	30 mg	(7.5–45 mg)
Olanzapine	15 mg	(2.5–70 mg)	15 mg	(2.5–60 mg)
Quetiapine	200 mg	(25–2400 mg)	200 mg	(25–800 mg)
Risperidone	3 mg	(0.5–100 mg)	2.75 mg	(1–6 mg)
Pregabalin	200 mg	(25–1200 mg)	175 mg	(100 - 600 mg)

### Comorbidities and somatic medication

Somatic comorbidities of all patients monitored by the AMSP program were documented starting in 2007, resulting in a subset of 207,054 patients. The diagnoses of arterial hypertension, diabetes mellitus, congestive heart failure, obesity, and chronic venous disease, which are associated with an increased risk of severe edema, were significantly more common in the edema patients (*n*= 127, 44.1%) than in the total monitored population (*n*= 22,142, 10.69%). Of these comorbidites, essential hypertension was the most common diagnosis with 26 cases (20.5%) within the edema patients and 15,494 cases (7.48%) within the entire group. Comorbid obesity was also more prevalent among patients with edema (13 cases, 10.2%) than in the total study population (1622 cases, 0.78%). The remaining somatic comorbidities can be found in Table 5. The drugs used to treat somatic condition in patients with psychotropic-drug-induced edema and the aforementioned comorbidities summarized in groups can be found in Table 6. In particular, antihypertensive drugs, e.g., angiotensin-converting enzyme inhibitors (ACE-Is.), angiotensin II receptor blockers (ARBs), beta-blockers, and calcium channel blockers (CCBs) were frequently used (*n*=53). Among these, beta-blockers were used most commonly, with 25 prescriptions. Antidiabetic drugs were used in nine cases, with metformin being used most frequently (*n*=7).

**Table 5:** Comorbidities of all patients and those suffering from edema since 2007

Comorbidity (ICD-Code)	All patients, *n* (% of 207054)	Edema patients, *n* (% of 127)
Hypertension (I10.)	15494 (7.48%)	26 (20.47%)
Obesity (E66.)	1622 (0.78%)	13 (10.24%)
Diabetes mellitus (E11.)	4734 (2.28%)	9 (7.09%)
Heart failure (I50.)	689 (0.33%)	4 (3.15%)
Venous diseases (I87.)	73 (0.04%)	4 (3.15%)
All	22142 (10.69%)	56 (44.09%)

**Table 6:** Somatic medication of patients with edema since 2007

Drugs	Prescription frequency *n* (% of 127)
Beta blockers	25 (19.69%)
ARB	10 (7.87%)
CCB	9 (7.09%)
Antidiabetics	9 (7.09%)
ACE-I	9 (7.009%)
Digitalis glycosides	2 (1.57%)

*ACE-I* Angiotensin-converting enzyme inhibitors, *ARB* Angiotensin II receptor blockers, *CCB* Calcium channel blockers

**Table 7:** Edema in drugs that were prescribed less than 5000 times

Substance	Cases of edema *n*
Agomelatine	3
Analgesics	3
Mianserin	3
Cardiovascular agents	2
Gastrointestinal medications	2
Nortriptyline	2
Paliperidonpalmitate	2
Ziprasidone	2
Zotepine	2
Amitriptylinoxide	1
Buproprion	1
Gabapentin	1
Additives	1
Lipid-lowering drugs	1
Maprotiline	1
Moclobemide	1
Oxcarbazepine	1
Paliperidone	1
Prothipendyl	1
Sertindole	1
Thioridazine	1
Tranylcypromine	1
Vitamins	1
Zuclopenthixol	1
Zuclopenthixol acetate	1

## Discussion

We examined the frequency and concomitant circumstances of the occurrence of severe edema among 462,661 psychiatric inpatients treated with psychotropic drugs between 1993 and 2016. We defined edema as a severe ADR if it lasted more than 1 week and affected legs, arms, face, or multiple body parts, and in any case in which diuretics were used to treat edema. Under these well-defined criteria, we determined an incidence of psychotropic drug-induced edema of 0.50‰. This result differs from the data reported in registration studies, which indicate a higher incidence. However, these studies also include non-severe presentations of edema such as mild ankle edema, resulting in a more generous definition of ADR (European Medicines Agency 2004, 2008, 2010, 2014, 2022).

### Drugs

We did not identify an increased risk of drug-induced edema for any of the psychotropic drug groups, suggesting that it is individual drugs, rather than drug groups, that carry a significant risk of severe edema.

Within our study, mirtazapine was the psychotropic drug implicated in the highest number of edema cases. Mirtazapine-induced edema has mainly been described in case reports (Sullivan (2001); Anttila and Leinonen (2001); Lahdelma and Bruin (1998)). Peripheral edema has been listed as an ADR with a reported incidence of of 2.4% (Food and Drug Administration 1997), while some authors suggest an incidence of about 1% (Lahdelma and Bruin (1998); Anttila and Leinonen (2001)). Both of these incidences are significantly higher than the incidence we found (i.e., 0.60‰). Although the pharmacodynamic properties of mirtazapine have been extensively studied (Fawcett and Barkin 1998), it is not fully understood how they contribute to edema. As mirtazapine does not block 5-HT2 receptors, although discussed as a fundamental pathophysiologic cause, this mechanism does not adequately explain mirtazapine’s propensity to induce edema (Ng et al. 2003). Additionally, mirtazapine has a comparatively low affinity for H1-receptors and muscarinic acetylcholine receptors, which are also associated with drug-induced edema (Ng et al. 2003). Consequently, further studies are needed to elucidate the underlying pathomechanism.

The present study indicated that pregabalin carries a particularly high risk of causing severe edema (0.15%). However, this incidence is considerably lower than the findings of other research groups. Edema has been found to occur in 5.6–8.6% of patients treated with pregabalin [Toth (2014); Freynhagen et al. (2015); European Medicines Agency (2004)]. Baldwin and colleagues (2015) analyzed 13 clinical trials evaluating the efficacy and safety of pregabalin in generalized anxiety disorder and reported that the treatment with pregabalin therapy was discontinued by patients in 0.5% of cases due to peripheral edema. However, the overall incidence of pregabalin-induced edema was low (less than 3%). Elderly patients (Freynhagen et al. 2015) and females (Largeau et al. 2022) appear to be more susceptible to pregabalin-induced edema.

A study considering cases of edema under gabapentinoids reported to the French Pharmacovigilance Center suggests a mechanism of action similar to that of CCBs. Both drugs have a strong affinity for the alpha-1 subunit of voltage-dependent calcium channels resulting in a decreased myogenic tone and causing concentration-dependent vasodilation (Largeau et al. 2022). It remains unclear, whether the edema-causing properties of pregabalin are dose-dependent. While Calandre and colleagues (2016) report a dose–reponse relationship for edema, the 28 studies examined in the meta-analysis of Zaccara and colleagues (2011) did not suggest a dose-dependent effect. However, it must be mentioned that the results of the aforementioned studies encompass patients receiving pregabalin for various indications, including psychiatric and non-psychiatric conditions (except for Baldwin and colleagues 2015), such as neuropathic pain, fibromyalgia, and epilepsy. This diversity in patient populations may limit direct comparability of results. In our study, we did not observe a clear dose–response relationship. In fact, among the group of edema cases, the median dosages were lower than in the total monitored group.

### Sex

Our study revealed that women were four times more likely to develop psychotropic drug-induced edema than men. The higher risk of edema in women has been previously reported in association with other drugs, such as CCBs (Semel et al. 2010). Several factors may contribute to this gender difference, including heightened body awareness in women (Messerli (2002); de la Sierra (2009)). Furthermore, venous insufficiency and associated venous stasis are more common in women than in men (Lohr and Bush 2013), which can favor the development of edema (Largeau et al. 2021).

### Age

Older age is associated with an increased risk of edema due to changes in lymphatic contractility, decreased pump efficiency, and a higher risk of microvascular disease and hyperpermeability (Largeau et al. 2021). Greil and colleagues investigated the incidence of ADRs under psychopharmacotherapy in relation to age. They compared psychotropic drug use and the occurrence of ADRs in hospitalized patients in Switzerland younger than 60 to those older than 60 years and found that psychotropic drug-induced edema was more common in older patients (Greil et al. 2013). Similar trends have been observed with other drug classes, such as CCBs (Fogari et al. 2000) and gabapentinoids (Semel et al. 2010). The result of the present study only partially confirm the relationship between higher age and drug-induced edema. While the incidence of psychotropic drug-induced edema is lowest in those younger than 30 years, it is higher in those 30–60 years of age than in those older than 60 years. However, Greil and colleagues did not find an age relationship for allergic skin reactions as ADR (Greil et al. 2013). Since an allergic genesis of edema has also been postulated (Chen and Chou 2004) and the exact mechanisms of drug-induced edema are not fully understood, this aspect should also be considered.

### Comorbidities and somatic medication

The occurrence of drug-induced edema is not necessarily limited to one trigger, but may be favored by a combination of concomitant conditions. The fact that diseases, such as diabetes, chronic venous insufficiency, or obesity, have a negative influence on the development of edema has already been shown [Ely et al. (2006); Wu et al. (2017)]. Within our study, almost half of the patients with drug-induced edema also suffered from at least one of these conditions, with obesity and arterial hypertension playing a particularly significant role. While hypertension was reported in 26 cases and congestive heart failure in four patients with severe edema, prescriptions of antihypertensive drugs were recorded in 40 cases in our study. The difference in these numbers may, in part, be attributed to the possible underreporting of comorbidities. In addition, certain classes of drugs, such as beta-blockers, have indications beyond hypertension and congestive heart failure, such as migraine prophylaxis and glaucoma treatment (Fumagalli et al. 2020). In hypertension, both the disease itself and its progression to congestive heart failure, as well as the treatment with drugs, such as CCBs, beta-blockers, or alpha-agonists, are associated with peripheral edema [Domenic (2003); Besharat et al. (2021)].

On the other hand, the use of ACE inhibitors, an ARB with CCB therapy, or thiazides appears to reduce the incidence of edema [Messerli (2002); Weir et al. (2001)]. However, the fact that discontinuation of imputed psychotropic drugs led to symptom relief in most cases supports the presumption that edema was primarily related to the use of psychotropic drugs rather than the antihypertensive drugs.

Among antidiabetic drugs, insulin and thiazolidinediones are known to cause edema (Sunder et al. 2003). In our study, these drugs played only a negligible role, as thiazolidinediones were not used at all and insulin was used in only one case. Metformin, however, was used in seven patients with drug-induced edema. Interestingly, metformin may reduce the risk of edema by decreasing capillary permeability (Valensi et al. 1995). In general, it is important to consider comorbidities and preexisting drug regimes when edema occurs during treatment with psychotropic drugs as these factors can contribute to the complexity of the condition and its management.

### Management of drug-induced edema

The favorable outcome observed in the majority of the severe edema cases in our study, with symptoms fully resolving in over 80% of cases, suggests that appropriate treatment measures, such as dose reduction or discontinuation of the triggering drug or the use of a diuretic, are effective treatment options. At the end of the observation period, symptoms remained unchanged in only three cases. Due to the multifactorial nature of edema, a precise medical history, clinical examination, and, if necessary, further diagnostic workup, should be performed (Trayes et al. 2013). In the treatment of drug-induced edema, it is recommended to discontinue or reduce the dose of the implicated drugs if possible [Trayes et al. (2013); Tamam et al. (2002)], as it was done in most of the cases in the present study. However, because dose-dependent effects are not consistently described for all drugs (e.g., pregabalin) [Calandre et al. (2016); Zaccara et al. (2011)], the efficacy of this measure in improving edema must be evaluated in each case.

The use of diuretics in the treatment of drug-induced edema can be effective in reducing fluid retention, but it’s essential to consider additional adverse effects associated with diuretic therapy. Read and colleagues investigated such a prescribing cascade in patients with gabapentinoid-induced edema in a cohort study and pointed out relevant diuretic-associated adverse effects, such as orthostasis, falls, urinary incontinence, and electrolyte imbalances (Read et al. 2021). This underscores the importance of a personalized and cautious approach to treatment, especially in older patients, who may already be at higher risk for developing edema as well as ADRs in general (Charlesworth et al. 2015) and are also more likely to be treated with diuretics (Read et al. 2021).

### Strengths and limitations

AMSP is a standardized pharmacovigilance program with structured methods of data reporting. The extensive 23-year observation period during which nearly half a million inpatients were monitored offers a unique opportunity to identify rare ADRs in a real-life setting. Due to the inpatient setting, AMSP gathers data on actual drug utilization and not only the prescription of psychotropic drugs as is often the case in outpatient-based studies. However, several limitations of the AMSP program must also be considered. As is common with pharmacovigilance programs, ADRs are often underreported. This may especially be the case for conditions such as edema, which might not always be recognized as drug-related. While AMSP has standardized methods for data reporting, variations in how different monitors assess and document ADRs across different hospitals can introduce inconsistencies in the data. Not all ADRs may come to the attention of drug monitors, and the precise documentation of ADRs may be time-consuming. Furthermore, AMSP only assesses severe ADRs in inpatients and therefore does not include ADRs occurring after discharge unless the patient is (re-)admitted due to the ADR. The identification of the specific drugs responsible for the ADR can be complicated by polypharmacy and underlying comorbidities. It can be challenging to determine whether an ADR is solely caused by a single drug or if it is the result of drug–drug or drug–disease interactions. Additionally, undisclosed or unrecognized comorbidities may also have contributed to the development of edema.

### Conclusion

Psychotropic drug-induced edema may severely impact a patient’s overall well-being and therefore could reduce adherence to treatment or necessitate the use of additional drugs to manage the condition. Our study revealed that when considering individual drugs, pregabalin had the highest incidence of edema. Several factors, such as sex, comorbidities, age, and somatic medication, may increase the risk of edema, highlighting the importance of considering these factors when treating patients with psychotropic drugs. While most cases of drug-induced edema showed favorable outcomes and could be managed with therapeutic interventions, such as dose reduction and drug discontinuation, more severe cases may require a comprehensive diagnostic workup and treatment. Fortunately, appropriate therapeutic interventions can substantially alleviate the symptoms.

## Data Availability

Data for this study cannot be made available due to personal, sensitive content such as basic sociodemographic patient characteristics and diagnoses.
